# From Chaos to Opportunity: Decoding Cancer Heterogeneity for Enhanced Treatment Strategies

**DOI:** 10.3390/biology12091183

**Published:** 2023-08-29

**Authors:** Alessandro Ottaiano, Monica Ianniello, Mariachiara Santorsola, Raffaella Ruggiero, Roberto Sirica, Francesco Sabbatino, Francesco Perri, Marco Cascella, Massimiliano Di Marzo, Massimiliano Berretta, Michele Caraglia, Guglielmo Nasti, Giovanni Savarese

**Affiliations:** 1 Istituto Nazionale Tumori di Napoli, IRCCS “G. Pascale”, Via M. Semmola, 80131 Naples, Italy; mariachiara.santorsola@istitutotumori.na.it (M.S.); f.perri@istitutotumori.na.it (F.P.); m.cascella@istitutotumori.na.it (M.C.); m.dimarzo@istitutotumori.na.it (M.D.M.); g.nasti@istitutotumori.na.it (G.N.); 2 AMES, Centro Polidiagnostico Strumentale srl, Via Padre Carmine Fico 24, 80013 Casalnuovo Di Napoli, Italy; monica.ianniello@centroames.it (M.I.); raffaella.ruggiero@centroames.it (R.R.); roberto.sirica@centroames.it (R.S.); giovanni.savarese@centroames.it (G.S.); 3 Oncology Unit, Department of Medicine, Surgery and Dentistry, University of Salerno, 84081 Baronissi, Italy; fsabbatino@unisa.it; 4 Department of Clinical and Experimental Medicine, University of Messina, 98122 Messina, Italy; mberretta@unime.it; 5 Department of Precision Medicine, University of Campania “L. Vanvitelli”, Via Luigi De Crecchio 7, 80138 Naples, Italy; michele.caraglia@unicampania.it

**Keywords:** cancer heterogeneity, genetics, epigenetics, mutations

## Abstract

**Simple Summary:**

Cancer is a complex illness marked by aberrant cellular behaviors and genetic variances, resulting in tumor heterogeneity that presents challenges in cancer prognosis and treatment. This review underscores the significance of comprehending tumor heterogeneity, offering perspectives on its attributes and the intricacies involved in its quantification. By emphasizing the need for effective therapies targeting tumor heterogeneity, this work contributes to raising awareness about this elusive characteristic of cancer.

**Abstract:**

Cancer manifests as a multifaceted disease, characterized by aberrant cellular proliferation, survival, migration, and invasion. Tumors exhibit variances across diverse dimensions, encompassing genetic, epigenetic, and transcriptional realms. This heterogeneity poses significant challenges in prognosis and treatment, affording tumors advantages through an increased propensity to accumulate mutations linked to immune system evasion and drug resistance. In this review, we offer insights into tumor heterogeneity as a crucial characteristic of cancer, exploring the difficulties associated with measuring and quantifying such heterogeneity from clinical and biological perspectives. By emphasizing the critical nature of understanding tumor heterogeneity, this work contributes to raising awareness about the importance of developing effective cancer therapies that target this distinct and elusive trait of cancer.

## 1. Introduction

Cancer, a complex disease characterized by aberrant cell proliferation, migration, invasion, and survival [1], exhibits heterogeneity across multiple dimensions, including genetic, epigenetic, transcriptional, and phenotypic variations [2,3]. This heterogeneity stems primarily from genomic alterations driven by genetic mutations, copy number variations, and chromosomal aberrations, which underlie the fundamental hallmarks of cancer. These genetic changes cumulatively give rise to distinct subpopulations of tumor cells, each with diverse phenotypes that influence cell cycle dynamics, metabolic activity, and cell signaling pathways [4].

Tumor heterogeneity holds strategic advantages for promoting tumor growth, survival, and metastasis by augmenting the likelihood of acquiring mutations that confer selective advantages, such as drug resistance and immune evasion. Additionally, intra-tumoral heterogeneity contributes to varying responses to therapeutic interventions, as different subsets of tumor cells exhibit dissimilar sensitivities to treatments. Beyond direct genetic modifications, epigenetic factors play a contributory role in shaping heterogeneity during tumor development and progression. Epigenetic changes, encompassing DNA methylation, histone modifications, and non-coding RNA expression, significantly affect gene expression regulation without altering the DNA sequence itself. These epigenetic modifications are pervasive in cancer, culminating in the suppression of tumor suppressor genes and activation of oncogenes [5,6]. The understanding of tumor heterogeneity holds immense potential to revolutionize cancer diagnosis and treatment by enabling the identification of subpopulations of cells responsible for tumor progression and drug resistance.

This review aims to provide valuable insights into tumor heterogeneity as a fundamental feature of cancer, presenting readers with crucial information regarding this issue. Additionally, the study delves into the challenges associated with measuring and quantifying tumor heterogeneity, shedding light on often overlooked aspects such as vascular heterogeneity. By emphasizing the critical nature of comprehending tumor heterogeneity, this work underscores the importance of developing effective cancer therapies that target the unique and elusive characteristics of cancer.

## 2. Cancer Heterogeneity: Definitions

Intra-tumoral heterogeneity refers to the genetic diversity present within a single tumor. The existence of distinct subclones within a tumor can give rise to variations in tumor growth, response to therapy, and the development of drug resistance. For instance, different subclones within a tumor mass may carry distinct driver mutations, leading to a more aggressive phenotype and a poorer prognosis [7].

On the other hand, inter-tumoral heterogeneity refers to the genetic diversity observed among tumor masses in different patients who have tumors of the same type. This heterogeneity can stem from disparities in the underlying mutations driving tumor development or differences in the microenvironment where the tumors originate. As an illustration, breast cancer is recognized as a heterogeneous disease, comprising various subtypes characterized by distinct molecular profiles and clinical outcomes [8]. These different breast cancer subtypes exhibit diverse driver mutations and respond differently to therapies, underscoring the significance of inter-tumoral heterogeneity in disease management.

Temporal heterogeneity describes the genetic diversity that emerges over time within a single tumor or between different tumors in the same patient. This heterogeneity can arise from the accumulation of additional mutations in cancer cells as the disease progresses, or from the selection of different subclones due to therapy or other environmental influences [9].

Spatial heterogeneity encompasses the genetic diversity existing across different regions of a tumor in the same patient [10]. Intra-tumoral heterogeneity can be considered a form of spatial heterogeneity. This heterogeneity can arise from variations in the tumor microenvironment, such as differences in oxygen and nutrient availability or the presence of immune cells. Spatial heterogeneity can also result from disparities in the underlying mutations driving tumor growth in different regions of the tumor.

It is important to note that, although not entirely accurate but for the sake of simplicity, the term “genetic” used to describe tumor heterogeneity also encompasses epigenetic factors.

## 3. Angiogenic Heterogeneity of Tumors

Angiogenesis and vascular diversity play a pivotal role in the initiation and progression of tumors. Neo-angiogenesis, the process by which new blood vessels are formed from pre-existing ones, is crucial for tumor growth and the spread of cancer cells. Falkman has made significant contributions to the study of neo-angiogenesis in tumors, shedding light on the ability of tumors to stimulate the development of new blood vessels through the secretion of pro-angiogenic factors such as vascular endothelial growth factor (VEGF) and basic fibroblast growth factor (bFGF) [11]. Falkman’s research also highlights the dynamic and heterogeneous nature of this phenomenon.

**Figure 1:**
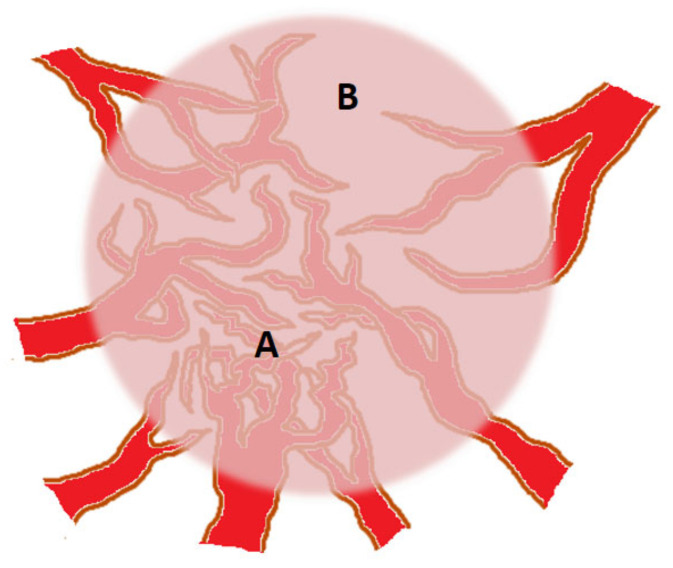
The circular and pink shadow indicates a tumor mass. Tumor blood vessels can be heterogeneous in terms of density, with areas of higher density (A) and lower density (B) within the same tumor mass. This can significantly influence tumor growth and response to treatments. The effectiveness of chemotherapy is diminished in region (B), where the transportation and cellular absorption of drugs by tumor cells are inferior compared to region (A). Additionally, within (B), regions are dominated by quiescent tumor cells that are less responsive to the effects of chemotherapeutic agents. These cells exhibit a reduced sensitivity and demand lower levels of oxygen and nutrients to sustain their survival.

The primary objective of this section is not to provide an exhaustive account of all the factors and molecular mechanisms involved in tumor neo-angiogenesis, but rather to emphasize the contribution of vascular heterogeneity to tumor heterogeneity. As the tumor mass expands, it exhibits increasing morphological heterogeneity, resulting in diverse densities of neo-angiogenesis within the tumor [12] (illustrated in Figure 1).

Furthermore, in certain areas, besides the typical induction of endothelial cells to sprout and initiate angiogenesis through pro-angiogenic factors [13], phenomena like vascular mimicry (where tumor cells form vessel-like structures themselves) prevail [14]. In other areas, trans-differentiation of cancer stem cells into endothelial cells occurs [15], while in some regions, the tumor undergoes necrosis due to inadequate nutrient supply [16]. These areas result in reduced pressure on adjacent regions, induce an immune-suppressive environment, and ultimately promote the survival of the residual neoplastic population, typically at the periphery of the tumor mass. Furthermore, the expression of pro-angiogenic factors varies among tumors, leading to differences in vascularization and oxygenation.

These differences can have significant clinical implications, as tumors with lower vascularization may exhibit reduced responsiveness to therapies targeting blood vessels [17]. For instance, the efficacy of anti-VEGF therapies, aimed at inhibiting tumor blood vessel growth, is limited by the presence of heterogeneity in VEGF expression. Imaging techniques such as contrast-enhanced ultrasound (CEUS) [18] and magnetic resonance imaging (MRI) [19] have been employed to assess tumor vascularization. These techniques enable the visualization of blood flow and quantification of vascularization within tumors.

In a study involving breast cancer patients, CEUS was utilized to compare the vascularization levels among different breast cancer subtypes. The findings revealed that the more aggressive triple-negative subtype exhibited a lower degree of vascularization in comparison to other subtypes [20].

Intra-tumoral vascular heterogeneity pertains to the existence of distinct vascular patterns within various regions of the same tumor. Some regions may demonstrate a high density of blood vessels, while others may be avascular. This heterogeneity can be influenced by factors such as variations in subclonal secretion of pro-angiogenic factors, tumor size, and anatomical location [21]. Recent studies employing single-cell genomics have shown that intra-tumoral vascular heterogeneity is associated with poor prognosis in several types of cancer [22,23,24,25].

The presence of intra-tumoral vascular heterogeneity can significantly impact the delivery of drugs to the tumor. Regions with a high density of blood vessels are likely to experience more effective drug delivery compared to avascular regions [26,27,28]. This observation holds crucial implications for the design of drug delivery strategies, as it may be necessary to target drugs specifically to regions with high vascular density in order to maximize their effectiveness.

Several studies have identified potential biomarkers of angiogenic and vascular heterogeneity that could be utilized to predict treatment response and patient outcomes. For instance, the expression of VEGF has been linked to poor prognosis in various types of cancer, including breast and lung cancer [29,30,31,32].

Further research is imperative to gain a deeper understanding of the mechanisms underlying angiogenic and vascular heterogeneity, develop accurate measurement techniques, and devise more effective therapies for tumors exhibiting heterogeneous vascularization.

## 4. Consequences of Heterogeneity on Prognosis and Response to Therapy

**Figure 2:**
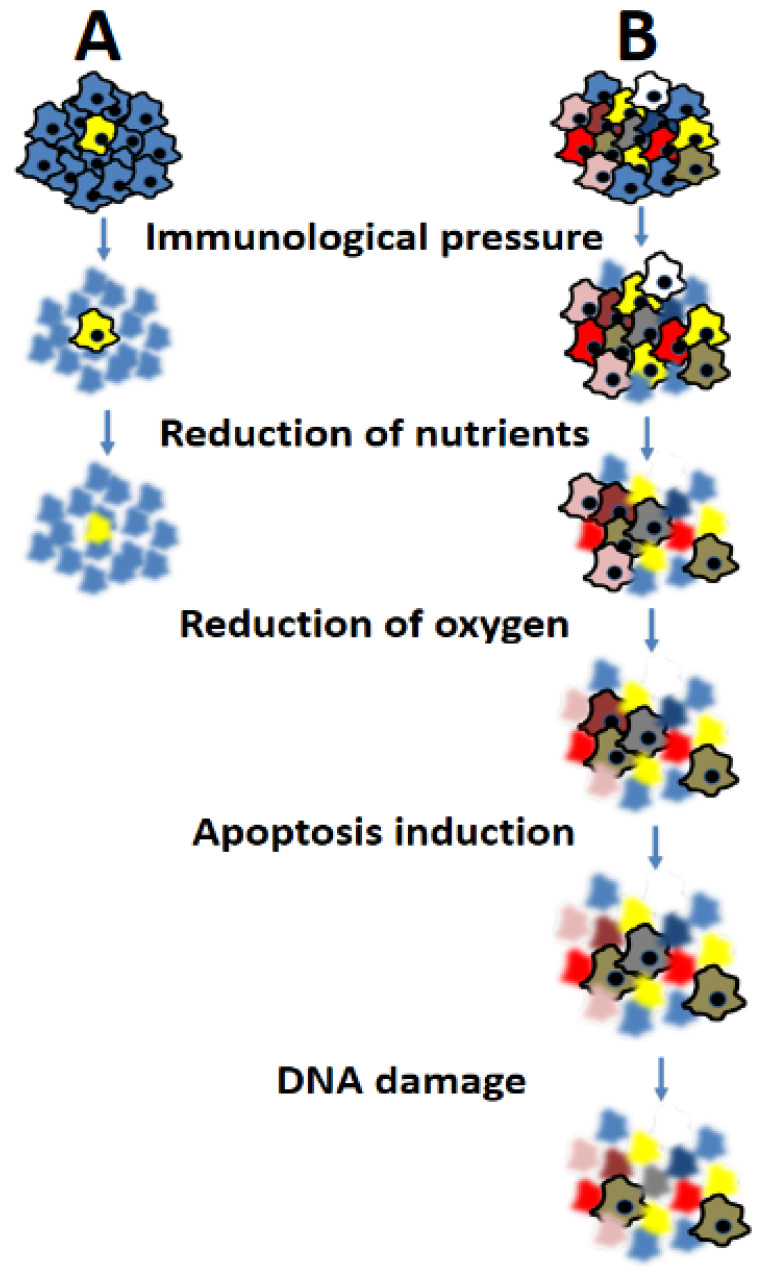
A tumor mass with a genetically more homogeneous cellular population (**A**) is evolutionarily disadvantaged compared to a more heterogeneous one (**B**). The cellular mass in (**B**) can withstand and overcome all obstacles that limit its growth.

The presence of heterogeneity among cancer cells poses a significant challenge, particularly because it allows tumors to evade treatment following an initial response. This is a direct consequence of the cancer cell’s remarkable genetic plasticity throughout its evolutionary history. The pursuit of enhanced survival in an increasingly hypoxic and nutrient-poor environment, compounded by the immunological hostility encountered at each stage, necessitates the selection of clones with genotypic and phenotypic traits that ensure their survival as shown in Figure 2.

Not all cells are likely to survive or give rise to progeny. In this context, cancer establishment may not be a frequent occurrence, although its exact frequency cannot be quantified. It is reasonable to assume that the relative risk of cancer formation and progression is significantly lower than the number of unsuccessful attempts. Once a tumor mass is established and clinically detectable, it becomes a highly heterogeneous genetic entity, functioning as an ideal evolutionary system. Pivotal studies have provided substantial evidence that cancer heterogeneity plays a pivotal role in shaping the tumor microenvironment (TME), including the immune response to cancer [33,34]. The TME plays a pivotal role in influencing cancer heterogeneity, and it adds further complexity to the issue as an extrinsic factor. It consists of a complex and dynamic ecosystem comprised of various cellular and non-cellular components, including immune cells, fibroblasts, endothelial cells, extracellular matrix (ECM), and soluble factors [35]. The interactions between tumor cells and the components of the TME are instrumental in shaping the genetic and phenotypic plasticity observed in cancer [36]. One of the key players in the TME is the immune compartment, which includes various immune cells such as T cells, B cells, natural killer cells, dendritic cells, and macrophages [35,36]. The crosstalk between tumor cells and immune cells can have profound effects on cancer heterogeneity. The immune system’s selective pressure can lead to the emergence of tumor cells with immune evasion capabilities, resulting in the expansion of subclones that are less recognizable by the immune system. This phenomenon, known as “immunoediting”, can drive tumor evolution and profoundly shape cancer heterogeneity [37,38]. Moreover, the stromal compartment within the TME also plays a crucial role in influencing cancer heterogeneity. Cancer-associated fibroblasts (CAFs) are a major component of the stromal compartment and are involved in ECM remodeling and paracrine signaling. CAFs can promote tumor growth and invasion and contribute to therapy resistance by providing a supportive niche for tumor cells [39]. Additionally, the ECM composition and structure influence tumor cell behavior, migration, and invasion, contributing to the heterogeneity observed in cancer progression [40,41]. The spatial organization of tumor cells within the TME also contributes to cancer heterogeneity. Tumor cells can exhibit distinct phenotypes depending on their spatial location within the tumor mass (adjacency to blood vessels, necrotic zones, peri-neural areas, etc.), leading to spatial heterogeneity [42,43,44]. This spatial heterogeneity has important clinical implications, as it can influence treatment responses and disease progression.

The extent of cancer heterogeneity can influence treatment response and prognosis. A recent study examined the genetic diversity of non-small cell lung cancer (NSCLC) tumors through whole-exome sequencing. The researchers discovered that tumors with higher genetic diversity had a poorer prognosis and shorter progression-free survival [33]. Similarly, the genetic diversity of colorectal cancer tumors is linked to chemotherapy resistance [34]. In these studies, intra-tumoral heterogeneity was primarily assessed using algorithms associated with “divergent” somatic mutation rates (i.e., mutations not shared by all analyzed lesions within the same patient). It is crucial to further explore this concept, as non-shared mutations can refer to both mutations that differ within different regions of the same tumor mass and those comparing the primary lesion with metastatic lesions. Epigenetic changes can also contribute to cancer heterogeneity. Modifications such as DNA methylation or histone acetylation can influence gene expression, resulting in the development of distinct subclones with different phenotypes [45,46,47]. In fact, DNA methylation patterns in diffuse large B-cell lymphoma (DLBCL) tumors, along with high levels of methylation heterogeneity, are associated with a worse prognosis and shorter overall survival [48].

**Figure 3:**
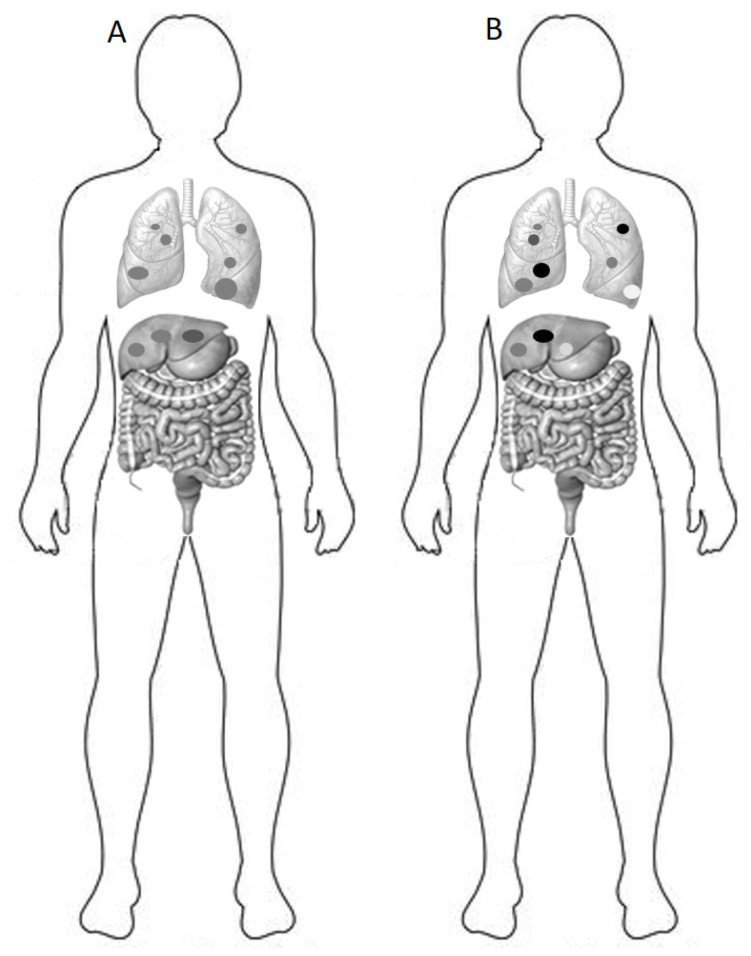
Metabolic heterogeneity is visualized using lesions with varying shades of gray, where darker shades indicate higher glucose uptake. It is assessed using positron emission tomography (PET) imaging with the glucose analog tracer 18F-fluorodeoxyglucose (FDG) and reveals differences in the metabolic behavior of cancer cells within different patients patient (**B**) exhibits more pronounced heterogeneity in metastatic lesions compared to (**A**). This heterogeneity suggests the presence of distinct metabolic characteristics in certain regions or subpopulations of cancer cells, which can impact tumor growth, aggressiveness, and treatment response. Genetic alterations, microenvironmental conditions, and cellular adaptations are among the factors that contribute to the development of metabolic heterogeneity.

Although the primary focus of this review is not an exhaustive exploration of tumor metabolism, it is crucial to underscore the clinical and therapeutic implications. Within the intricate landscape of cancer biology, metabolic heterogeneity emerges as a pivotal concept. Specifically, diverse glucose uptake and consumption patterns in cancer cells can significantly influence both tumor progression and therapeutic responses. Notably, positron emission tomography (PET) imaging can identify metabolic heterogeneity in tumors (as illustrated in Figure 3). Tumors with pronounced heterogeneity in glucose uptake are linked to poorer overall survival and reduced chemotherapy responsiveness [49,50,51,52,53].

Furthermore, in recent years, considerable interest has been gained by radiomics. Radiomics is a field in medical imaging that involves the extraction and analysis of extensive quantitative data from radiographic images, such as CT scans or MRI, in order to characterize tumors. Radiomics not only holds potential in terms of predicting therapy outcomes but can also be useful in predicting the degree of tumor heterogeneity in genetic terms [54,55,56,57]. 

Preclinical investigations conducted on animal models and in vitro systems have established the influence of cancer heterogeneity on the efficacy of immunotherapy. For instance, the existence of diverse fractions of slow-cycling cells, commonly known as “quiescent” cancer cells, within the tumor mass can impede the effectiveness of immune checkpoint inhibitors [58]. Additionally, cancer heterogeneity can also encompass the variability in the recruitment of distinct populations of immune and immuno-regulatory cells within the TME [59]. The composition of the TME, including the presence of immune-suppressive cells such as regulatory T cells and myeloid-derived suppressor cells, can further impact the response to immunotherapy [60,61]. Furthermore, the evaluation of tumor heterogeneity through next-generation sequencing profiling serves as a significant predictor of the response to immune checkpoint inhibitors in patients with advanced melanoma [62]. Similarly, both intra-tumoral genetic and epigenetic diversity have been associated with a suboptimal response and unfavorable survival outcomes in patients with various solid tumors [63,64,65,66,67,68,69].

It is important to emphasize that genetic heterogeneity and expression of tumor antigens are not the same thing, nor are they necessarily associated. In other words, tumor mutational burden (TMB) [70], typically the number of mutations per megabase of the tumor genome, and microsatellite status (MicroSatellite Status) [71], which are associated with increased expression of tumor antigens, do not represent the concept of genetic heterogeneity, at least not with the same therapeutic implications [72,73,74]. In fact, genetic and epigenetic heterogeneity, unlike high TMB or microsatellite instability, are associated with greater resistance to chemotherapy and immunotherapy [63,64,65,66,67,68,69]. This is a fascinating field, a puzzle that requires time and research to be solved.

## 5. Mathematical Models Used to Quantify Cancer Heterogeneity

Several mathematical models have been proposed to quantify cancer heterogeneity and provide a quantitative assessment of the genetic diversity within a tumor [75,76,77]. The Shannon [78], Simpson [79], and Gini [80] indices are three widely utilized models in cancer research. By offering a quantitative measure of the genetic diversity within a tumor, these models have been employed to predict treatment outcomes, identify potential therapeutic targets, and measure intra-tumoral and inter-tumoral heterogeneity. In this paragraph, we provide a concise description of these mathematical models used to assess cancer heterogeneity. The Shannon index quantifies the diversity of a population by considering its species richness and evenness. Originally used to quantify biodiversity in ecology, it has been adapted for application in cancer research. In the context of cancer, the Shannon index quantifies the genetic diversity within a tumor based on the frequency of different mutations present. The formula for the Shannon index is H = −Σpi ln pi, where pi represents the proportion of cells carrying a specific mutation, and ln denotes the natural logarithm. The index ranges from 0 (indicating no diversity) to a maximum value that depends on the number of unique mutations present. The Simpson index is another diversity measure commonly employed in ecology, which has also been adapted for cancer research. It provides an assessment of the genetic diversity within a tumor. Unlike the Shannon index, the Simpson index considers the dominance of different mutations within the tumor. The Simpson index is calculated using the formula D = Σ(pi)^2^, where pi represents the proportion of cells carrying a specific mutation. This index ranges from 0 (maximum diversity) to 1 (no diversity), with higher values indicating a more dominant clone. The Gini index, on the other hand, is a measure of inequality commonly employed to quantify income distribution. In the context of cancer research, the Gini index has been adapted to assess the inequality in the distribution of mutations within a tumor. The formula for the Gini index is G = (2/n) Σ(pi − qi)(I − 1/2), where pi represents the proportion of cells carrying a specific mutation, qi is the cumulative proportion of cells carrying mutations up to and including i, and n is the total number of mutations. The index ranges from 0 (maximum diversity) to 1 (no diversity), with higher values indicating a more unequal distribution of mutations.

These indices have been utilized in predicting treatment outcomes and identifying potential therapeutic targets in various types of cancer [78,79,80,81,82,83]. While there are many other models available, providing a comprehensive explanation of each is beyond the scope of this review. For further information on these models, we refer readers to other relevant manuscripts [84].

However, a profound reflection is necessary. These models provide a momentary snapshot of tumor heterogeneity without considering the dynamic changes in tumors over time. This factor introduces complexity to the modeling process, which may necessitate the use of more advanced models [85], theories (such as chaos theory) [86,87], technologies (e.g., single-cell sequencing) [88], and algorithms (such as artificial intelligence) [89] in the future. Although this study does not delve into these aspects, it is crucial to explain to the reader that tumor heterogeneity is a dynamic system that exhibits exponential sensitivity to its initial conditions. These systems follow deterministic and biological laws but can also exhibit empirical randomness in their evolution (hence the relevance of chaos theory as a possible approach to studying tumor heterogeneity). The temporal dimension is integral to the variability of all systems, and the cancer phenomenon distorts the time variable in a biological evolutionary sense that deviates from what is considered “normal”. DNA mutations are a fundamental component of human evolution [90,91], suggesting that cancer may arise as a consequence of mutations, which are a naturally occurring phenomenon that creates genetic variability in humans. Equation 2 NcµP represents a model of evolution based on the number of chromosomes (K = 2 Nc in a diploid population), the census population size (Nc), the mutation rate (µ), and the probability that a mutation spreads and becomes fixed (P) [92]. This model is applicable to sites subject to selection. The rate of neutral evolution, R, can be calculated using this equation. Time plays a crucial role in this model, as µ represents the rate of new mutations occurring in a gene within the period between two generations, assuming no back mutations. Beneficial mutations resulting in stable gain-of-function mutations typically require thousands of generations to manifest their fitness effects.

The typical mutation rate in individuals, i.e., the number of non-synonymous somatic mutations accumulated per base-pair/gene during their lifetime, remains unknown but is expected to be extremely low [93]. Eukaryotic cells have developed efficient mechanisms to repair DNA damage caused by cosmic radiation and other mutagens over millions of years. To estimate the mutation rate within a short period, many researchers employ rapidly dividing organisms like Escherichia coli or yeast to track mutational events (both harmful and beneficial) induced by environmental factors, drugs, and radiation [94]. During the early stages of most malignant cancers, the “time” variable is distorted in favor of rapidly accumulating favorable mutations for growth and spreading. This pathological alteration of genes related to DNA repair (e.g., p53, MMR genes, BRCA, BAP1, etc.) increases the likelihood of mutations occurring, coupled with an individual’s allelic “susceptibility” to cancer. When analyzing genetic profiles of primary malignant neoplasms that develop into poly-metastatic spread, frequent alterations are observed in genes responsible for maintaining DNA integrity. These aberrations are necessary for cancer cells to manipulate the time variable in their favor and evolve rapidly at the expense of the host. Mutations in these genes provide cancer cells with a high adaptive evolutionary power, enabling them to exploit various neoplastic phenomena such as the angiogenic switch, immune system evasion, epithelial-to-mesenchymal transition, migration, invasion, and more. This mutational plasticity represents genetic trajectories that lead to the adaptation and high fitness levels of tumor cells. Recently, the use of next-generation sequencing (NGS) techniques has revealed that the TMB in malignant cells surpasses that of normal cells, highlighting the increased mutation rate occurring in cancer cells [95].

## 6. Measuring Tumor Heterogeneity: Challenges and Perspectives

There is an increasing interest in the development of novel methodologies to measure and quantify cancer heterogeneity (Table 1). As mentioned earlier, one direct approach involves assessing the genetic heterogeneity of tumors through sequencing techniques, although the technical details of these methods are beyond the scope of this review. Furthermore, an alternative approach, which we can refer to as “indirect,” involves evaluating vascular or metabolic heterogeneity, which can be examined using imaging techniques such as MRI or PET.

It is worth repeating, however, that the most direct and reliable way to measure tumor heterogeneity is by evaluating cancer genetics. Our first and most intuitive definition is that tumor heterogeneity refers to the genetic diversity within a single tumor. In other words, the presence of different subclones within a tumor can result in differences in tumor growth, response to therapy, and the development of drug resistance. We emphasize, given the complexity of the topic, that TMB is not an indicator of tumor heterogeneity. While a high mutation rate might lead to the assumption of more heterogeneous tumors, it does not necessarily correspond to the concept of genetic heterogeneity. Paradoxically, a “homogeneous” genetic instability possessed by the tumor, due, for example, to DNA repair mechanism alterations, exposes the tumor to increased production of neo-antigens and therefore greater immunological visibility [70,71,72,73,74]. 

Presently, the most scientifically honest response is that we lack uniform and dependable tools to evaluate tumor heterogeneity across various cancer types. It remains largely elusive. It is certainly a challenge for the future that could change our approach to oncology and treatments. 

It is now clear that tumor heterogeneity, not only between different histotypes which is easier to understand, but also within the same histotype and tumor mass, can heavily influence treatment response. Two examples can be considered: the lack of response to imatinib in non-GIST *c-kit* mutated tumors and the biochemistry of *KRAS* p.G12C mutations in colorectal cancer. In the first case, imatinib exerts its therapeutic effect by binding to the ATP-binding site of the hyper-activated mutated c-kit protein in exon 11, preventing its activity and inhibiting downstream signaling pathways [96]. In the second case, inhibitors (sotorasib and adagrasib) bind to the inactive GDP-bound form of KRAS p.G12C, locking it in an inactive state, unable to activate intracellular signals [97]. However, it is true that not all solid tumors with *c-kit* exon 11 or *KRAS* p.G12C mutations respond brilliantly to therapy. Indeed, the response rate to *KRAS* p.G12C mutations in colorectal cancer, a disease that has been extensively studied, exhibits considerable variability and does not exceed 7% [98].

The answer, albeit simple, lies in tumor heterogeneity. In both cases, numerous other genetic and epigenetic molecular partners can modify the activity of c-kit and KRAS, thereby influencing the initial response or the development of secondary resistance. Some of these partners for c-kit include Grb2, Grb7, Grb10, Shc, Gab2, Src, and for KRAS, Grb2, RASGEF, and SOS1 [99,100,101]. The “lateral” heterogeneity of these factors can certainly influence and condition different responses both within the same tumor and, even more so, across different tumors. Understanding and measuring this heterogeneity could significantly enhance anti-tumor treatments.

Building upon this concept, moreover, not only do we lack a reliable and precise tool to measure tumor heterogeneity, but it also leads to difficulties in characterizing tumors, as different regions of a tumor may exhibit different genetic profiles. This can introduce sampling bias, where the genetic information obtained from a single biopsy may not accurately represent the entire tumor. Traditional approaches to cancer diagnosis and treatment rely on bulk tumor analysis, which obscures the intra-tumoral heterogeneity. Recent advancements in single-cell sequencing technologies have facilitated the identification of genomic and transcriptional heterogeneity within tumors. These technologies allow for the analysis of individual cells, providing a high-resolution view of the genetic and transcriptional landscape of tumors [102,103]. Single-cell sequencing has uncovered previously unrecognized subpopulations of tumor cells and identified rare subclones that may play crucial roles in tumor growth and progression [104]. In the following sections, we delve more comprehensively into the methodological advantages and limitations of single-cell characterizations.

Notably, the genetic characterization of matched primary and metastatic lesions in patients with metastatic colorectal cancer is a valuable approach that can serve as a model for studying cancer heterogeneity. In two previous studies using NGS-based whole exome sequencing in patients with metastatic colorectal cancer, we observed that patients with a less aggressive disease course exhibited higher levels of genetic concordance in terms of mutational events between the primary tumor and metastases [105,106]. Genetic concordance, defined as the percentage of mutations in shared coding gene regions between metastases and the primary tumor, reflects genetic heterogeneity. It is reasonable to assume that increased genetic heterogeneity leads to decreased concordance between metastases and the primary tumor. Furthermore, in patients undergoing adjuvant chemotherapy, the concordance between the primary tumor and metastatic recurrence was generally lower. Our hypothesis is that chemotherapy may induce genetic remodeling in descendant cells, which subsequently develop into metastases. This phenomenon suggests that genetic heterogeneity and plasticity play functional roles in the evolution and progression of cancer. Regarding TMB, we have consistently emphasized that while it does not directly correlate with genetic heterogeneity, it provides an estimate of the cellular population’s propensity to mutate. Lastly, it is worth noting that the mutational status of p53, as we have seen earlier, can be correlated with genetic heterogeneity. However, to delve deeper into its critical role in triggering genetic plasticity, we direct readers to other scientific works for further information [107].

## 7. Exploring Cancer Heterogeneity: Challenges and Prospects in Model Systems

Advanced genetic tools, such as genetically engineered mouse models (GEMMs), offer the capability to precisely activate oncogenes and deactivate tumor suppressors in a controlled spatial and temporal manner. This strategy facilitates the initiation of tumors through specific genetic events, thereby enabling the investigation of cancer heterogeneity. Employing this approach permits researchers to functionally assess the oncogenic properties and resistance to therapeutic interventions of genetic and genomic alterations identified in human tumors. Illustrative instances include the induction of oncogenic KRAS activity in colorectal and lung cancers [108], the amplification of HER2 in breast cancer [109], and the targeted inactivation of p53 across various cancer types [110,111,112]. It is noteworthy that the advancement of the Cre-Lox strategy and CRISPR/Cas9 methodologies has significantly hastened the progression of murine models of human malignancies [113,114].

The pivotal scientific question within the scope of this work is: Can these non-human models be valuable for investigating tumor heterogeneity? Indeed, certain facets of the trajectory of human cancer are arduous, if not unfeasible, to replicate in models. This encompasses considerable tumor size and the protracted timeline of tumor evolution. Nonetheless, a notable advantage of murine models in this context lies in their capacity to monitor neoplastic cells from cancer initiation to the emergence of metastases. In effect, even with the recurrence of biopsies, only a negligible proportion of human tumors can be scrutinized, whereas complete murine tumors can be conveniently procured. Consequently, theoretically, this stands as a substantial methodological benefit. However, one of the challenges encountered in reproducing non-human models is precisely the extent of genetic heterogeneity. To elucidate further, murine tumors display a diminished degree of genetic heterogeneity (primary vs. metastatic tumors) due to the absence of environmental mutagens and the controlled living environments of the mice utilized in these investigations, which also exhibit genetic similarity [108,109,110,111,112,113,114,115,116]. 

Therefore, the scientific community has undertaken efforts to enhance the reliability of murine models. Far from providing an exhaustive exposition of this topic, it is worth comprehending methodologically the approaches to achieve this goal. One approach to simulate the human scenario involves subjecting GEMMs to the same carcinogens suspected or known to induce cancer in humans. For instance, chemicals present in cigarette smoke have been employed [117]. Another genetic strategy to more closely replicate the heterogeneity observed in human tumors within mouse models involves manipulating genes associated with DNA repair or genome stability [118,119,120]. To mimic the heterogeneity seen in cancer, these DNA repair alterations could be combined with other oncogenic events in mouse models.

As previously discussed, the rapidly expanding array of single-cell methodologies for assessing cellular status at the DNA, RNA, and protein levels with increasing precision provide potent tools in these models to track tumor heterogeneity at the tissue context level over time and space. Without delving into the intricacies of all lineage-tracing techniques, it is crucial to acknowledge that it is feasible to track the fate of a specific clone of tumor cells (e.g., cells with a particular driver mutation) that have been “tagged” with an inheritable enzyme involved in emitting a traceable signal (typically a fluorescent protein) over time [121]. An analogous approach to lineage-tracing studies, offered by GEMMs, involves the capability to selectively eliminate a specific subset of cells within tumors to interrogate their role in tumor maintenance and progression. Such lineage-ablation experiments commonly utilize a system where a suicide gene, such as the diphtheria toxin receptor (DTR), is placed under the control of gene regulatory elements that exclusively mark a cell type of interest [122]. Mouse cells exhibit insensitivity to diphtheria toxin (DT), an exceptionally potent cytotoxic peptide, due to the absence of the diphtheria toxin receptor (DTR) expression. This phenomenon facilitates the targeted elimination of specific cell subpopulations by systemic administration of DT. Beyond strategies reliant on suicide genes, certain cell types possess distinctive surface markers that can be leveraged for the eradication, marking, or monitoring of cells through the utilization of monoclonal antibodies [123,124]. Furthermore, as previously mentioned, non-human models facilitate comparisons between the genetics of primary tumors and nearly complete metastatic ones. The evolving ability to track tumor genetics using increasingly sophisticated and less invasive techniques will undoubtedly transcend the limitations of non-human models in depicting cancer heterogeneity. Lastly, noteworthy are the “organoids” as models for studying cancer heterogeneity. Organoids hold significant value as they are capable of replicating certain histopathological, genetic, and phenotypic attributes of tumors [125,126,127]. In fact, in contrast to conventional tumor cell lines and xenografts in mice (human tumor cells implanted into immunodeficient mice), which often lack representation of the TME and display limited diversity, organoids provide a more comprehensive platform. Through co-culturing with non-tumor cells, they can simulate TME–cell interactions and offer a closer emulation of the original tumor characteristics. While challenges persist in standardizing organoid culture techniques and defining successful culture parameters, their potential to replicate tumor heterogeneity and their utility in high-throughput drug screening for precision medicine position them as a promising model for cancer research. 

## 8. Treating Genomic Heterogeneity: Theoretical Pathways for Cure

The aim of this review is to raise awareness of how tumor heterogeneity represents the most significant underlying oncological challenge, influencing the entire spectrum of tumor phenomena, from biological behavior to treatment response. Although its measurement and monitoring remain challenging, what are the therapeutic strategies to address tumor heterogeneity? Tumor heterogeneity poses a substantial obstacle in developing effective treatment strategies for cancer patients. This paragraph examines two ideal approaches for treating tumor heterogeneity: the sequential approach and the combination approach. Both approaches possess their strengths and limitations, which are discussed below. 

The sequential approach involves administering a single drug (with or without chemotherapy) initially, based on known genetic alterations in the tumor, followed by reassessment of tumor genetics and subsequent modifications to the treatment. This approach acknowledges the dynamic nature of tumor heterogeneity and aims to target emerging clones with new genetic alterations. The sequential approach allows for adaptation and adjustment of treatment based on the evolving tumor landscape. By re-evaluating the tumor genetics, clinicians can identify emerging clones with additional driver alterations and select subsequent therapies accordingly. This approach considers the unique genetic profile of each patient’s tumor, enabling tailored treatment selection based on individual tumor characteristics. Furthermore, administering targeted drugs sequentially can prevent unnecessary exposure to multiple drugs simultaneously, potentially reducing the risk of adverse effects and improving tolerability. However, the sequential approach necessitates iterative cycles of tumor assessment, which can result in delays in initiating optimal treatment and limit the opportunity for rapid disease control. Additionally, there is a risk of “incomplete targeting” as this approach relies on identifying and addressing newly emerging genetic alterations. Consequently, there is a possibility of overlooking key driver alterations or therapeutic targets that are present from the onset, potentially leading to suboptimal treatment outcomes.

The combination approach involves the simultaneous administration of multiple targeted drugs, each targeting different key driver genetic alterations identified in the tumor. This approach aims to address the heterogeneity by simultaneously attacking multiple pathways or clones within the tumor. The combination approach offers the potential to address multiple genetic alterations and target diverse subclones within the tumor simultaneously. This broader coverage increases the likelihood of achieving a more comprehensive treatment response. Furthermore, combining drugs with different mechanisms of action may lead to synergistic effects, enhancing treatment efficacy and potentially overcoming resistance mechanisms. By targeting various clones upfront, the combination approach may achieve early control of the tumor, potentially reducing the risk of disease progression. However, combining multiple drugs can increase the risk of adverse effects and toxicity, potentially impacting patients’ quality of life and necessitating careful monitoring and management. The combination of drugs may eventually lead to complex interactions, including pharmacokinetic and pharmacodynamic interactions, making dose optimization and scheduling challenging. Finally, it should be noted that the use of multiple drugs simultaneously could significantly increase treatment costs, potentially limiting accessibility and affordability for some patients.

## 9. Potential Strategies for Advancing Precision Treatment of Cancer Heterogeneity

Tumors possess the capability to adapt and mold their genetic landscape in response to therapeutic interventions and unfavorable microenvironmental conditions. This necessitates the development of treatment approaches that are both dynamic and adaptive. One potential strategy involves the continuous monitoring of genetic changes in cancer using single-cell sequencing and genetic characterization technologies. These robust tools enable the evaluation of the genomic profiles of individual tumor cells, thereby revealing insights into clonal evolution and identifying subpopulations with distinct molecular characteristics. Single-cell sequencing is a groundbreaking technique that permits the examination of the genetic material of individual tumor cells. This approach offers a glimpse into the genome, transcriptome, and other multi-omics at the single-cell level. The objective of this study is not to delve into the specifics of single-cell whole-genome amplification techniques (scWGA). For certain methods such as MALBAC, eMDA, LIANTI, SISSOR, and META-CS, further reading of other works is necessary [128,129,130,131,132]. It is important to understand that these techniques share a commonality in that the cellular sample can originate from primary tumors or metastatic sites. Furthermore, it is crucial to perform proper cell isolation and dissociation through mechanical or enzymatic methods to break down the tissue into individual cells. Subsequently, the isolated single cells are individually captured using microfluidic devices or plates. The DNA of the captured cells undergoes scWGA, and the resulting genomic DNA libraries are then subjected to high-throughput sequencing. The sequencing data obtained for each individual cell contains valuable information about gene expression, mutations, copy number variations, and other genomic features. Single-cell sequencing enables the identification of distinct cell populations within a tumor, offering a comprehensive view of its heterogeneity. By tracking genetic changes in individual cancer cells over time, single-cell sequencing can uncover the evolutionary trajectory of tumors and the acquisition of critical characteristics. It is worth emphasizing that the isolation of single cells represents a pivotal stage in single-cell sequencing techniques, ensuring that the genetic material obtained accurately represents individual cells rather than a mixture of cells. Given that formalin-fixed paraffin-embedded (FFPE) tissues are a globally prevalent method for preserving clinical samples, it is essential to highlight that several methods have been developed to extract viable genetic material from FFPE samples for single-cell analysis. These methods encompass laser capture microdissection (LCM), where a laser precisely targets and captures specific cells of interest, facilitating subsequent single-cell sequencing. Alternatively, there is single-nucleus sequencing, a process that entails isolating and sequencing the nuclei instead of whole cells. The limitations inherent in single-cell sequencing primarily stem from the constraint of low input material, which heightens vulnerability to technical noise and biases. Moreover, variations in cell viability, morphology, and capture efficiency can significantly influence the precision of downstream analysis, particularly when examining infrequent cell subpopulations. 

**Figure 4:**
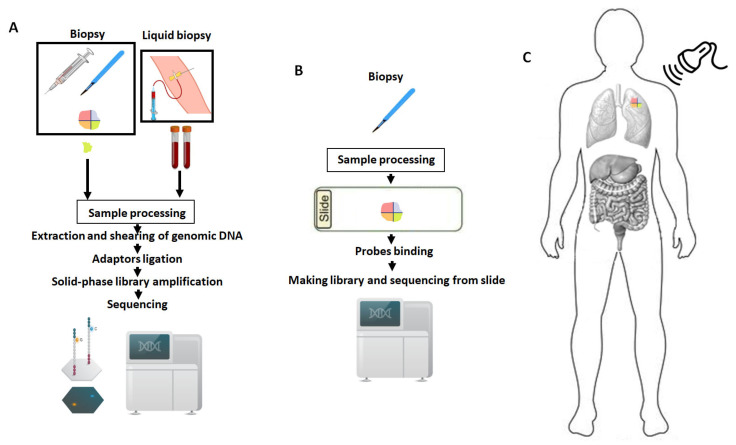
In (**A**), the main methodological steps that enable next-generation sequencing (NGS) from solid or liquid tumor biopsies are exemplified. In (**B**), utilizing nanostring technology reduces the number of steps as the assessments are performed directly on the tissue. In the future, it is conceivable that biopsies may not be required, and biological macromolecules will be read directly from the patient (**C**).

However, an ideal precision treatment against cancer heterogeneity requires the integration of innovative technologies and multi-omic data from different time points and spatial locations within the tumor. Technology advancements will assume a crucial role in this context, and a promising approach known as “nanostring technology” has emerged [133,134,135] (Figure 4). In comparison to NGS (Figure 4A), this innovative method enables direct “reading” of individual molecules (through a plethora of very small probes called “nanostrings”), including RNA, and proteins in the tissue context (Figure 4B). An innovative example is the nanoString GeoMx^®^ Digital Spatial Profiler (DSP) designed for the comprehensive profiling of proteins and RNA within constrained tissue samples [136,137]. Even if it has no single-cell resolution, this cutting-edge technology provides insights into tumor characteristics through spatial and temporal evaluations enabling the simultaneous examination of multiple biomarkers in tissue samples preserved either by freezing or formalin-fixed paraffin embedding. The application of distinctive indexing oligos enables the direct and accurate quantification of targeted proteins and RNA molecules. A key advantage of the DSP technique is its non-destructive nature, allowing subsequent studies to be conducted using the same tissue slides after the profiling process.

However, in the future, we have the potential to extend our knowledge from partial insights, as current nano-analyses in solid biopsies are limited to a small tumor tissue sample, to a comprehensive 3-D and time-related context. This revolution is no longer confined to science fiction; it will become feasible through the adoption of cutting-edge technologies that harness the principles of “quantum technology” to explore molecules with unprecedented levels of detection sensitivity. In this transformative context, physics, in conjunction with the aforementioned disciplines, will assume a pivotal role. While substantial effort and time will be required, the implementation of a quantum-based sequencing method could stand as a significant advancement in elucidating the distinct signals generated by varying electronic structures of DNA nucleotides [138,139]. Notably, this approach might unlock opportunities for physicians to scrutinize the genetic and molecular compositions of tumor lesions using non-invasive modalities (sounds, scans, etc.) (Figure 4C). In a hypothetical scenario, employing this approach could grant researchers unparalleled insights into the intricate dynamics of cancer heterogeneity.

## 10. Conclusions

This work highlights the critical nature of comprehending and affording tumor heterogeneity in developing effective cancer therapies. Targeting this unique and elusive characteristic of cancer must become a priority for future anti-cancer research.

## Figures and Tables

**Table 1:** Examples of modalities to assess cancer heterogeneity.

Heterogeneity Category	Assessment Technique	Output
Genetic	Next-generation sequencing	Genetic variants, mutations, copy number alterations [63,64,65,66,67,68,69]
Epigenetic	Next-generation sequencing	DNA methylation patterns, histone modifications [3,4,5,6,45,46,47,48]
Angiogenic	Ultrasound, magnetic resonance imaging (MRI)	Density and distribution of blood vessels [17,18,19,20,21]
Metabolic	Positron emission tomography	Uptake of glucose [49,50,51,52,53]
Morphological	Radiomics	Scores that combine multiple morphological features [54,55,56,57]
Morphological and genetic	Spatial transcriptomics thorugh in situ hybridization and single-cell RNA sequencing	Genetic characterization of single cell in its biological context [21,22,23,24,25]

## Data Availability

Not applicable.
